# Smoking cessation in emergency departments: a reality check

**DOI:** 10.1007/s11739-026-04528-2

**Published:** 2026-09-24

**Authors:** D. Campagna, F. Cucuzza, C. Russo, J. A. Putra, P. Caponnetto, G. R. M. La Rosa, U. Prosperini, S. Agrawal, R. Polosa

**Affiliations:** 1 Center of Excellence for Acceleration of HArm Reduction (CoEHAR), University of Catania, Catania, Italy; 2 Department of Clinical and Experimental Medicine, University of Catania, Catania, Italy; 3 UOC MCAU, University Teaching Hospital “Policlinico-S.Marco”, University of Catania, Catania, Italy; 4 Ashford and St.Peter’s Hospitals, NHS Foundation Trust, Chertsey, UK; 5 Panti Rapih Hospital, Universitas Negeri Yogyakarta, Yogyakarta, Indonesia; 6 Department of Educational Sciences, Section of Psychology, University of Catania, Catania, Italy; 7 Institute of Psychiatry Unit, AOU Policlinico G. Rodolico-San Marco, University of Catania, Catania, Italy; 8 UOC Pronto Socco-OBI Presidio Ospedaliero Taormina, ASP Messina, Messina, Italy; 9 Institute of Lung Health, Glenfield Hospital, Leicester, UK; 10 University Hospitals of Leicester NHS Trust, Leicester, UK; 11 Faculty of Medicine and Surgery, “Kore” University of Enna, Enna, Italy; 12 Centre for the Prevention and Treatment of Tobacco Addiction (CPCT), University Teaching Hospital “Policlinico-S.Marco”, University of Catania, Catania, Italy

**Keywords:** Emergency department, Smoking cessation, Brief intervention, E-cigarette, Pharmacotherapy

## Abstract

Emergency Departments (EDs) see a high prevalence of people who smoke, many of whom present with acute smoking-related conditions (e.g., myocardial infarction, stroke, pneumonia, and asthma/COPD exacerbations). The immediacy of illness creates a strong “teachable moment,” increasing receptiveness to risk communication and behavior change. Evidence indicates that pairing brief counseling with point-of-care therapeutic support and structured follow-up improves abstinence, although effects vary with local infrastructure, product availability, and staffing models. Despite this promise, scale-up is often constrained by economic barriers, limited staffing and training, and weak integration of cessation workflows into routine ED practice. The aim of this article is to provide a reality check on ED-based smoking cessation by synthesizing evidence on effectiveness, feasibility, cost-effectiveness, and safety across health-system contexts, and by outlining pragmatic delivery models and research priorities. Embedding low-friction, clinically governed cessation bundles within EDs has the potential to convert acute presentations into durable quit attempts and reduce smoking-related harm at scale, though this population-level impact remains a hypothesis to be tested. Future work should prioritize pragmatic and implementation trials with biochemically verified outcomes, integration metrics, equity impacts, and cost-utility analyses to guide sustainable adoption across diverse settings.

## Background

Leveraging the Emergency Department (ED) as a venue for smoking intervention is strategic and necessary [1], given the high smoking prevalence among ED attendees and the frequency with which smoking contributes to presenting complaints [2–5]. Although relatively few formal programs have been embedded within EDs to date (e.g., targeted initiatives in the US and the UK), interest is rising as evidence accumulates that an acute health crisis, and the fear it provokes, can prompt a powerful teachable moment for quitting [6].

Designing effective pathways in EDs is challenging. The ED mandate prioritizes time-critical care; throughput pressures and crowding constrain behavioral assessment and counseling. Even when clinically relevant, preventive services (smoking cessation, alcohol screening, immunizations) are often treated as non-essential by busy ED staff during acute encounters. Consequently, many smokers who pass through EDs receive no cessation advice. For a sizeable subset, however, the ED is their only point of contact with the healthcare system, underscoring the importance of brief interventions, pharmacotherapy initiation, and referral to ongoing support.

This opinion article offers a reality check on ED-based smoking cessation. We assess how EDs, often the only contact point for underserved patients, can meaningfully reduce smoking-related harm and inequities. We synthesize evidence on effectiveness, feasibility, cost-effectiveness, and safety of ED-based interventions, and contrast trial results with routine practice, highlighting operational constraints. We also outline knowledge gaps to clarify what works, for whom, and under which operational conditions in the ED setting.

## Methods

This article is framed as an opinion piece. A literature search was conducted in PubMed from inception to June 2026, using the terms "smoking cessation" and "tobacco use," combined with "emergency department" and/or "emergency service," "brief intervention," and "tobacco control." Priority was given to randomized-controlled trials and systematic reviews with clear relevance to ED-based smoking cessation; case reports, conference abstracts, and unpublished or non-peer-reviewed literature were not considered.

For each selected study, key data (study design, population, intervention, comparator, and primary outcome) were extracted directly from the published article and summarized in Table 1.

**Table 1:** Summary of the included studies

Study	Design	Population	Intervention vs comparator	Primary outcome	Key results	Strengths	Limitations
Neuner et al. 2009 [48]	RCT	1,044 adult smokers, urban ED (Berlin, Germany); median age 30 years; 56% unmotivated, 32% ambivalent, 13% motivated to quit; race/ethnicity not reported; median 15 cigarettes/day	ED-initiated counseling (on-site, median 30 min) + up to 4 telephone booster sessions vs usual care	CO-verified 7-day abstinence at 12 months	No significant overall effect: 14.2% vs 11.3% (OR 1.31, 95% CI 0.91–1.89, p=0.15). Non-significant trend favoring intervention in motivated smokers (OR 2.19, 95% CI 0.98–4.89)	Pioneering European ED trial; large sample	Underpowered relative to original effect-size assumptions; substantial loss to follow-up (34.4% in both arms); biochemical validation limited to a small subsample
Bernstein et al. 2011 [44]	RCT	338 adult smokers (contemplation/preparation stage), urban ED (Bronx, NY); mean age 40 years; 52% female; 62% Hispanic, 22% African American; median 15 cigarettes/day	Enhanced Care (motivational interview + nicotine patches + follow-up phone call at 72 h + cessation brochure) vs Usual Care (cessation brochure alone)	Salivary cotinine and CO-verified 7-day abstinence at 3 months	No significant difference: 14.7% vs 13.2% (p=0.68). Tobacco-related ED diagnosis independently associated with quitting (OR 3.42, 95% CI 1.61–7.26)	Integrated multicomponent design	Single interventionist for both arms; enhanced "Usual Care" condition and assessment reactivity may have attenuated the between-group difference
Katz et al. 2013 [10]	Pre-post implementation trial	789 adult smokers (≥ 5 cigarettes/day) enrolled at two Iowa EDs, August 2008–August 2010; convenience sample	ED nurse-initiated 5A’s intervention (staff training, modified intake form, quitline referral, pharmacotherapy quick-orders) vs pre-intervention period (usual care, same sites)	7-day point-prevalence abstinence at 6 months	No significant difference, 6.8% (intervention) vs 5.1% (pre-intervention) (adjusted OR 1.7, 95% CI 0.99–2.9, p=0.054); significant improvement in nurse-delivered 5A’s composite score (1.47 vs 0.83) did not translate into significantly higher quit rates	Pragmatic two-site design with each site as its own control; validated 5A’s composite score; biochemical confirmation of self-reported abstinence	Reduced statistical power due to lower-than-expected enrollment/higher attrition; ~40% lost to 6-month follow-up; only ~50% of self-reported quitters provided saliva for biochemical confirmation; excluded light smokers (< 5 cig/day); single Midwestern state limits generalizability
Pelletier et al. 2014 [19]	Systematic review	13 ED-based studies (11 US, 1 Germany, 1 Turkey)	Various interventions (advice, counseling, MI, NRT)	Abstinence	Most studies (11/13) found no significant difference in abstinence between groups; only 2 (both MI-based) showed significant effect;	Structured critical appraisal; broad synthesis	No standardized control group across studies; mostly weak-to-moderate quality; predominantly self-reported abstinence
Lemhoefer et al. 2017 [62]	Systematic review and meta-analysis	11 RCTs total (7 from prior review + 4 newly retrieved), 1,392 participants in the newly retrieved studies; ED settings, multiple countries across America and Europe	ED-initiated tobacco control (on-site motivational interviewing/advice, often with booster calls and NRT) vs usual care/brief advice	7-day point-prevalence abstinence at 1, 3, 6, and 12 months	Pooled RR significant at 1 month (RR 1.49, 95% CI 1.08–2.05, p=0.01) and 3 months (RR 1.38, 95% CI 1.12–1.71, p=0.003); not significant at 6 months (RR 1.09, 95% CI 0.84–1.41, p=0.54) or 12 months (RR 1.26, 95% CI 1.00–1.59, p=0.05). Cumulative effect across all follow-ups: RR 1.40 (95% CI 1.06–1.86, p=0.02)	Multi-database search; formal risk-of-bias assessment; generalized linear mixed models accounting for between- and within-study heterogeneity; funnel plot showed no significant publication bias	Effect weaker in biochemically-validated studies only (RR 1.34 vs 1.40 overall); considerable heterogeneity in intervention components; one included study reported only as a conference abstract with insufficient data for bias rating
Cheung et al. 2018 [20]	RCT	1295 adult smokers, urban academic tertiary-care ED (Vancouver, Canada); mean age 40 years; 62% male; race/ethnicity not reported; light 50%, moderate 30%, heavy 21%	“Ask, advise, and refer” + referral to community counseling service (quitline/text/web) vs usual care (no referral, no counseling)	Self-reported 30-day abstinence at 12 months	No significant difference: 20.8% vs 18.1% (adjusted OR 1.60, 95% CI 0.87–2.94). Non-significant trend favoring intervention at all time points	Large sample (1,295 patients); pragmatic real-world design; blinded follow-up assessors; longitudinal ITT analysis with attrition sensitivity checks	Single-center; ~50% eligible not enrolled; usual-care arm partly contaminated (received "ask" step); repeated follow-up calls may have biased toward null; self-reported outcomes only
Castello et al. 2022 [47]	RCT	480 adult smokers, urban university hospital ED (Novara, Italy); mean age 44 years; 62% male; Italian population; mean 14 cigarettes/day	5A’s counseling by trained nurses + referral to smoking cessation center vs usual care (brief advice to quit only)	CO-verified point prevalence of tobacco-free patients at 6 and 12 months	No significant difference at 6 months (RR 1.04, 95% CI 0.58–1.87) or 12 months (RR 0.86, 95% CI 0.50–1.47). Counseling delivered to only 48% of intervention group (126/262)	RCT design with participant blinding to allocation; both ITT and PP analyses; embedded process evaluation (nurse survey) explaining implementation gaps	Underpowered -only 480 of the pre-specified target of 1,200 patients enrolled despite extending recruitment from 5 to 14 months; suboptimal screening/enrollment procedures (concentrated in a few months, run by an ad hoc junior researcher rather than trained ED staff throughout); low intervention fidelity, with nurses citing lack of time as the main barrier to delivering counseling
Pettit et al. 2022 [18]	Narrative review	ED settings (adults, pediatric, pregnant patients); international perspective (USA, UK, Germany, Italy, Scandinavia)	Multiple approaches (SBIRT, STIR, pharmacotherapy, counseling, quitline, e-cigarettes) without direct own comparison	Abstinence	Cessation is feasible and effective in the ED across adult, pediatric, and pregnant populations, though strategies remain poorly standardized despite the ED’s potential to address disparities and support national cessation goals	Broad multi-population, multi-country synthesis; practical pharmacotherapy reference table; identifies research gaps	Narrative, not systematic; no registered protocol or formal bias assessment; non-reproducible study selection
Pope et al. 2024 [16]		972 adult daily smokers, six NHS EDs (England and Scotland, UK); mean age 40 years; 62% male; 73% White British; median 15 cigarettes/day	Brief advice (up to 15 min) + e-cigarette starter kit + referral to local stop smoking services vs written information on stop smoking services only	CO-verified continuous abstinence at 6 months	Significant effect: 7.2% vs 4.1% (RR 1.76, 95% CI 1.03–3.01, p=0.038). Self-reported 7-day abstinence also significantly higher: 23.3% vs 12.9% (RR 1.80, 95% CI 1.36–2.38, p<0.001)	Large multicentre sample; objective primary outcome; robust randomization; pragmatic, replicable design; first ED trial testing e-cigarettes	Control arm received more than true usual care (CO testing, repeated questioning), likely inflating control quit rate; challenging CO verification uptake (~ 30% lost to follow-up; ~50% biochemical confirmation among self-reported quitters)

*cNMA* Component Network Meta-Analysis, *CO* Exhaled Carbon Monoxide, *ITT* Intention to Treat, *NRT* Nicotine Replacement Therapy, *MI* motivational interviewing, *PP* per protocol, *RCT* Randomized Controlled Trial, *SBIRT* Screening, Brief Intervention, and Referral to Treatment, *STIR* Screening, Treatment Initiation, and Referral

### Models of smoking cessation in the ED

Early models of intervention in the ED were centered on brief smoking cessation advice (e.g., Very Brief Advice) and printed materials (e.g., anti-smoking leaflets/flyers). These approaches are easy to deliver, but limited by low patient engagement (notably among adolescents) [7, 8], and inconsistent implementation due to ED time pressures and lack of interest among staff.

An additional strategy is the Ask-Advise-Refer (AAR) Model, in which clinicians ask about tobacco cigarette use, advise patients to quit, and refer them to external cessation services (e.g., Quitlines) [9]. The AAR model is simple and time-efficient for the ED context [10]; however, its effectiveness relies heavily on engagement with off-site follow-up, and the resulting quit rates remain modest [7]. Short and personalized adaptations (e.g., text messaging support) may improve outcomes [11].

Other evidence-based approaches include Motivational Interviewing (MI) [12] and the Screening, Brief Intervention, and Referral to Treatment (SBIRT) model [13]. However, their effectiveness depends on the availability of trained staff and consistent workflows, requirements that are difficult to sustain in crowded EDs.

Pharmacological treatments such as nicotine replacement therapy (NRT) and other FDA-approved medications remain a key component of smoking cessation in the general population, and their effectiveness increases when combined with behavioral support [14]. ED-initiated pharmacotherapy, combined with brief education and structured follow-up, can support an immediate transition away from smoking [15, 16].

Mobile apps, telehealth, and automated text messaging can deliver ongoing support after discharge, but their application in ED settings remains insufficiently explored and warrants dedicated evaluation [17].

### What the evidence tells us

A growing body of studies shows that ED-initiated cessation is feasible and often effective, but standardization is limited and most trials report 6–12-month abstinence, leaving long-term effectiveness uncertain. Outcomes are heterogeneous, reflecting differences in study design, implementation fidelity, and the health-system context, with notable variation by geography, available follow-up pathways, and access to pharmacotherapy and referral services.

In the United States, there is a general consensus on the usefulness and applicability of smoking cessation protocols already in EDs [18, 19]. Much of the early implementation has used AAR [20], MI-based brief interventions [19], and Electronic-Health Record (EHR) integrated referrals to Quitlines [21, 22]. These models demonstrate feasibility under study conditions but often report steep relapse and diminished effects beyond short follow-up. Smoking prevalence among US ED patients is high (approximately 30–40%) [23, 24], representing a three- to fourfold excess over the average US population prevalence, and thus conferring considerable reach on ED-based cessation interventions. Innovations include SMS programs, app-based support, and pharmacotherapy prescriptions at discharge [25, 26].

The same cannot be said in the European context, reflecting diverse funding and differing healthcare infrastructure [18]. The greatest progress has been in the Netherlands, and Scandinavia, supported by nationally funded cessation services [27–29] and, in the UK, pragmatic use of e-cigarettes within ED and funded community cessation pathways [16]. In resource-constrained EDs, funding, crowding and limited continuity often blunt impact despite sound clinical logic.

Non-pharmacological interventions (brief advice, MI) are effective, but providing concrete tools (NRT, e-cigarettes, pharmacotherapy) enhances effect sizes [14, 30]. Evidence from the general smoking-cessation population—not derived from ED settings—indicates that nicotine e-cigarettes are effective cessation aids. The August 2026 update of the Cochrane living review (80 RCTs, 29,861 participants) found high-certainty evidence that nicotine e-cigarettes produce higher quit rates than NRT at 6 months or longer (RR 1.61, 95% CI 1.23–2.12). However, data beyond 12–24 months remain limited, so the long-term effectiveness and safety of e-cigarettes for smoking cessation are still uncertain [31].

Nonetheless, real-world evidence from survey data aligns with trial data. In England’s Smoking Toolkit Study, increasing prevalence of e-cigarette use was positively associated with overall quit attempt rates and quit success [32]. In France, a nationwide cohort found that, among adult smokers, daily vaping is associated with a decrease in smoking level and an increase in smoking cessation attempts [33].

In the United States, longitudinal analyses of baseline smokers in the ADJUSST and PATH studies show that switching to regular e-cigarette use predicts subsequent smoking cessation and lowers relapse risk [34–36]. Notably, in PATH, even participants without plans to quit who adopted e-cigarettes achieved significantly higher cessation rates [37]. Overall, these population-level data consistently support e-cigarettes as an effective cessation aid, though long-term safety data remain limited and warrant continued surveillance.

### The CoSTED program: a current benchmark

The Cessation of Smoking Trial in the Emergency Department (COSTED) was a multicentre, parallel-group, randomized-controlled trial conducted across six EDs in the UK [16, 38]. Participants were randomized to brief advice + e-cigarette starter kit + referral to stop-smoking services versus written information alone. At 6 months, biochemically verified abstinence was higher in the intervention group (7.2%) than in the control group (4.1%) (RR = 1.76; 95% CI 1.03–3.01; p = 0.038). Self-reported abstinence was also higher in the intervention group (23.3% vs. 12.9%; RR = 1.80; 95% CI 1.36–2.38; p < 0.001), with no serious adverse events related to participation [16]. Longer-term follow-up suggests higher odds of remaining abstinent up to 18 months post-randomization [39]. Economic evaluation indicated cost-effectiveness by NICE thresholds [40], and process evaluation found the package acceptable and adaptable across ED contexts [41].

While the COSTED program suggests that an opportunistic, low-intensity package (brief advice plus an e-cigarette starter kit in EDs) can be clinically effective, cost-efficient, and feasible for broader implementation (reaching smokers who might not otherwise engage), several caveats temper interpretation. The absolute increase in biochemically verified abstinence at 6 months was modest. Moreover, the large gap between self-reported and verified quits (by expired CO) raises concerns about reporting bias. These factors warrant caution when extrapolating effectiveness beyond the trial context.

The favorable economic result depends on modeled assumptions (relapse, device costs, long-term QALYs) that could materially shift cost-effectiveness. Implementation feasibility in real-world ED settings also warrants caution: trial conditions likely benefited from dedicated research staff and controlled workflows that may not be replicable in routine practice. Moreover, the findings are context-specific to the UK, where vaping products are regulated and cessation services are widely available; generalizability to other health systems remains uncertain. Future studies should move beyond proof-of-concept to tease apart *what works* within the CoSTED package and *why*. A pragmatic, multi-site program should extend follow-up to 24 months to track continuous abstinence and relapse trajectories with biochemical verification to reduce misclassification. Equally important is real-world performance: studies should embed implementation metrics (reach, time-on-task, staff burden), equity analyses across socioeconomic groups, and cost-utility sensitivity to device pricing and relapse assumptions. Finally, cluster designs across diverse EDs (urban/rural; high-/low-resource) are needed to establish external validity and determine whether an ED-based screen–treat–refer pathway can deliver durable, equitable cessation at scale.

### Emerging evidence

Using e-cigarettes or other nicotine alternatives as cessation tools in the ED is a promising but still nascent strategy [18]. The UK CoSTED trial, the first RCT to initiate e-cigarette use in the ED, showed that provision of an e-cigarette starter kit at discharge, combined with brief advice and referral, produced higher biochemically verified 6-month abstinence than usual care [16]. Notably, over half of eligible patients consented, suggesting that the ED visit may represent a receptive context for cessation support [41].

Evidence also highlights pragmatic limits. Without structured follow-up (e.g., scheduled check-ins, refill access, titration of pharmacotherapy) and seamless hand-offs to community cessation services, outcomes remain modest, a pattern mirrored in U.S. studies despite differing health systems. A promising next step would be to test scalable pathways that begin in the ED, initiating cessation pharmacotherapy or, where appropriate, an e-cigarette as a harm reduction option, combined with brief MI-style counseling, automated e-referral, and scheduled 7–30-day contacts. Whether such a pathway can be sustainably implemented at scale remains to be established through pragmatic implementation trials.

Cytisine, a safe, effective and low-cost pharmacotherapy for smoking cessation, has recently been added to the WHO’s Essential Medicines List [42]. Given its affordability and favorable safety profile, cytisine should be evaluated as a first-line option in ED-initiated cessation programs, particularly in resource-limited settings where access to other pharmacotherapies such as varenicline may be restricted.

In addition to abstinence outcomes, implementation metrics (such as reach, uptake, time-on-task, and cost per quitter) should be systematically assessed. Embedding these measures allows programs to evaluate both clinical and operational performance. A standardized, light-touch model that integrates these metrics can maintain ED efficiency while significantly improving patients’ chances of sustained abstinence through practical, scalable intervention delivery.

### What works (and where): lessons from the UK, USA, and Europe

Evidence diverges by level of support. UK/US studies consistently show improved quit outcomes when brief counseling is paired with NRT or an e-cigarette starter kit and structured follow-up. By contrast, in many European settings, especially resource-constrained EDs, heavy workload, limited smoking cessation infrastructure, and weak follow-up capacity blunt effectiveness. Here, disappointing quit rates likely reflect a combination of factors, including delivery gaps, inadequate intervention intensity, lower patient readiness to quit, and implementation constraints. Without clear leadership, designated pathways, and accountability, ED-based cessation is unlikely to embed sustainably into routine acute care. Where those elements have been put in place, available evidence suggests that benefits may be reproducible, although further confirmation across diverse settings is needed.

The UK-led CoSTED trial [16] currently sets the methodological benchmark for ED smoking-cessation interventions (robust RCT design with biochemical verification at 6 months), yet it also faced substantial loss to follow-up and required notable resources. The positive CoSTED findings have stimulated interest in implementation beyond the original trial sites, and implementation resources have subsequently been developed to facilitate translation into routine ED practice. However, the extent, fidelity, and effectiveness of wider real-world implementation remain to be established.

US studies [3, 43, 44] also show solid study design, but they often rely on self-reported abstinence and face substantial loss to follow-up challenges. Moreover, their effectiveness and cost-effectiveness are best demonstrated in settings with integrated emergency systems with ready access to quitlines and EHR/IT infrastructure.

Across UK and US studies, a consistent pattern emerges: brief counseling + structured follow-up + point-of-care therapeutic support outperforms information-only approaches and aligns with WHO guidance [45]. External validity is strongest in integrated systems (e.g., UK NHS with e-cigarettes and stop-smoking services; US EDs with Quitlines and EHR e-referrals) and weakest where post-ED continuity is scarce.

In Italy, an ED in Novara embedded 5A’s counseling into nursing workflows, demonstrating organizational feasibility, but, without pharmacological/material support (e.g., NRT/e-cigarette kits), effects were limited [46]. This large Italian RCT (n=480) showed no significant benefit vs usual care at 6 months (RR 1.04; 95% CI 0.58–1.87) or 12 months (RR 0.86; 95% CI 0.50–1.47); only 18 counseled smokers attended a clinic and 12 completed treatment. Per-protocol effects were slightly larger, but the authors concluded the ED is not suitable for 5A’s counseling alone. These findings indicate that counseling without pharmacotherapies or e-cigarettes provision is insufficient in the ED.

Two additional European studies explored the feasibility and effectiveness of smoking cessation interventions delivered in ED settings. In Germany, a multicenter RCT (n = 1044 patients) testing ED-Initiated Tobacco Control intervention, which combined on-site counseling with up to four telephone booster sessions, did not increase 12-month abstinence rates versus usual care [47]. In Turkey, a brief, standardized 5-min ED smoking cessation counseling (with printed materials and referral contacts, plus a 1-month phone call for follow-up) was as effective as outpatient counseling, but referrals to dedicated cessation services were largely unsuccessful [48]. A more deepen analysis of these null findings reveals that delivery gaps are only part of the picture. In the German trial, the enrolled population was predominantly unmotivated to quit at baseline (56% unmotivated; only 13% motivated), a profile markedly different from CoSTED, where all participants were daily smokers approached in a receptive, crisis-exposed context. Moreover, neither the German nor the Turkish intervention included pharmacotherapy or a nicotine product, whereas CoSTED combined brief advice with an e-cigarette starter kit. These differences suggest that patient motivation, intervention content, and health-system support for post-ED follow-up are important contextual effect modifiers. In addition, a non-significant trend favoring the German intervention was observed among motivated smokers (OR 2.19; 95% CI 0.98–4.89), hinting that targeting cessation support toward higher-readiness subgroups may improve effectiveness even in resource-limited settings. Future trials should stratify by motivation stage and explicitly test whether the addition of pharmacotherapy or nicotine alternatives can rescue otherwise null counseling-only designs.

From the perspective of a lower- and middle-income country with very high smoking prevalence, such as Indonesia, adapting an ED-based smoking-cessation model presents both notable challenges and promising opportunities. Indonesian EDs commonly face staffing constraints, heavy caseloads, limited digital infrastructure for automated referrals, and inconsistent availability of NRT or regulated alternative nicotine products. These realities limit the direct transferability of high-income-country approaches that rely on EHR prompts and seamless referral linkages. At the same time, widespread mobile-phone access and expanding networks of community pharmacies and non-physician providers create a scaffold for light-touch pathways. Evidence suggests that Indonesian smokers are receptive to mobile-based cessation outreach (e.g., SMS/WhatsApp), and community pharmacists are an under-utilized but potentially willing workforce for cessation support, provided that targeted training and protocols are in place. A suitable pathway could embed nurse-led triage screening ("Ask about smoking"), immediate enrollment into an SMS/WhatsApp follow-up stream, and linkage to community partners, such as pharmacies [49], primary care centers [50], or vapeshops [51–53] that can assist with smoking abstinence. The potential role of harm-reduction outlets in this context warrants careful evaluation, taking into account local regulatory frameworks and potential conflicts of interest.

## Future directions

**Fig. 1:**
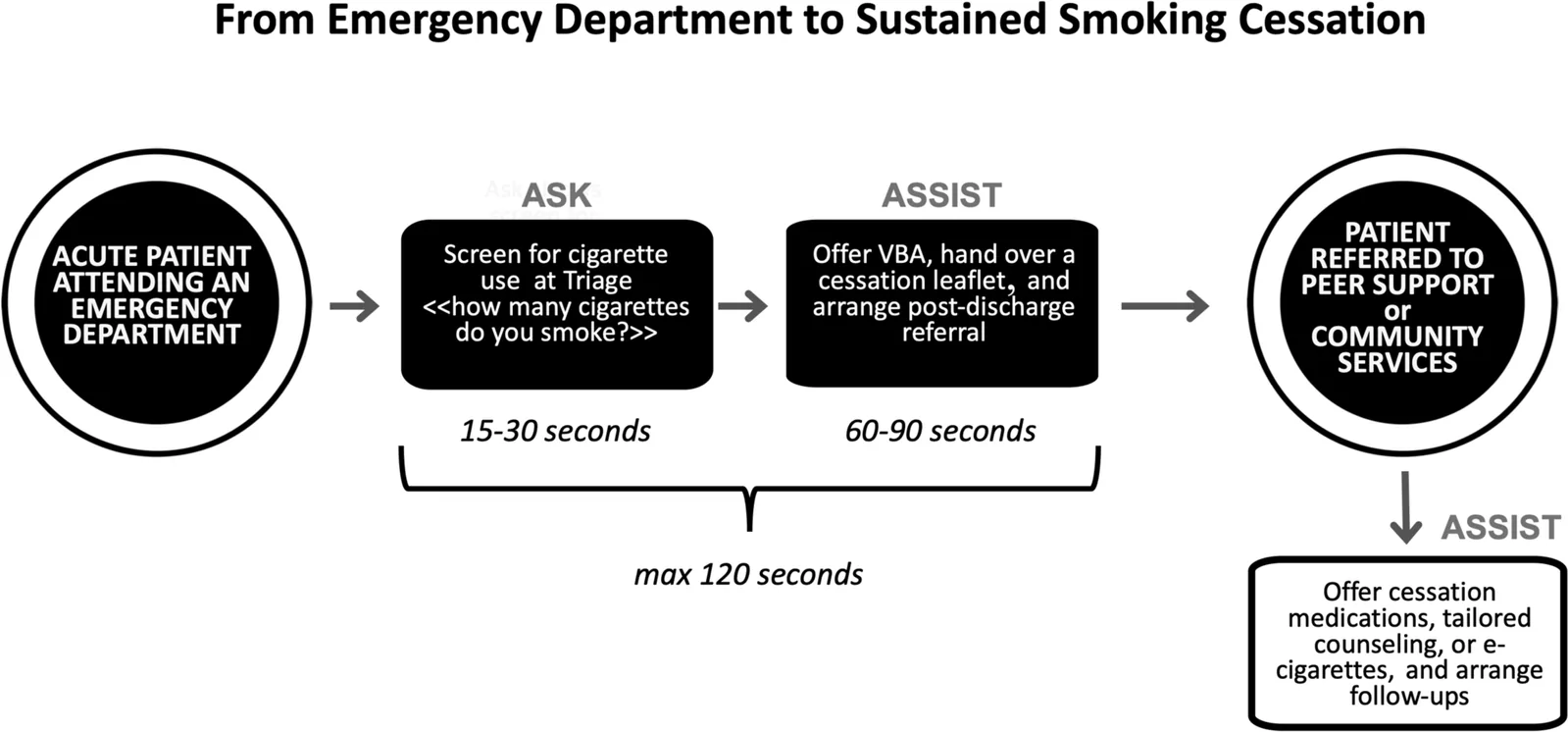
The diagram illustrates a proposed workflow for integrating smoking cessation support into Emergency Department practice. After identifying smokers during routine triage, clinicians provide very brief counseling (VBA) and, when appropriate, initiate a post-discharge referral to community services (i.e., quitlines, pharmacies, primary care centers, and community peer-support networks). Automated text reminders and early follow-up calls reinforce motivation and adherence

To move the field beyond proof-of-concept, we should pivot to operational science and build practical, standardized pathways that busy EDs can run with minimal disruption. The core idea is a pragmatic STIR model: screen at triage, initiate on-the-spot treatment (pharmacotherapy or e-cigarette) when appropriate and refer to cessation services [20], quitlines [54], or community “buddy” social networks [55] before discharge. With pre-packed materials and scripted prompts, the entire interaction can take two minutes or less while still creating a reliable bridge to follow-up care (see Fig. 1).

Evaluation must give equal weight to efficacy, feasibility, and cost-effectiveness. Future trials should report biochemically verified abstinence at 24 months, meaningful reduction, and safety, alongside real-world key performance indicators (e.g., reach, time per patient, pharmacotherapy uptake/adherence, linkage to community care, and impact on re-attendance). Because relapse is common, protocols should incorporate low-burden continuity: 7–30-day SMS/app check-ins, proactive quitline scheduling, community peer-support networks (though the latter represent emerging components that warrant dedicated evaluation in implementation trials), and partnerships with community pharmacies or harm-reduction services to facilitate access to starter therapies—an approach that warrants further evaluation in implementation research.

A persistent gap is the limited use of harm-reduction options. Offering nicotine options at discharge (e.g., NRT, e-cigarettes, where clinically appropriate and locally regulated) paired with light-touch digital follow-up may support harm reduction during the high-risk post-ED period, reduce relapse, and expands choice beyond pharmacotherapy to improve uptake and adherence [56]. This approach also keeps workflows low-burden through starter kits, automated follow-up, and clear community hand-offs.

Although the long-term health effects of e-cigarette use require further investigation, accumulating evidence suggests that these products are generally well tolerated, including among smokers with pre-existing cardiovascular and respiratory diseases. A large nationwide cohort study of smokers with coronary artery disease undergoing percutaneous coronary intervention reported lower risks of major adverse cardiovascular events among those who switched to e-cigarettes compared with those who continued smoking [57]. Similarly, prospective studies in patients with COPD and asthma have reported reductions in disease exacerbations and sustained respiratory improvements following substantial smoking reduction or cessation achieved through e-cigarette use [58, 59]. Recent systematic evidence has also identified favorable trends in vascular health among smokers who quit smoking or switched to e-cigarettes [60]. Nevertheless, while the emerging tolerability data are reassuring, further disease-specific data collected soon after acute respiratory and cardiovascular events are needed. It is important to note that the following tolerability and health-outcome data derive entirely from general clinical and observational cohorts unrelated to ED-initiated cessation programs; they inform the safety profile of e-cigarettes as a product class but provide no direct evidence about ED-based delivery models.

### Practical recommendations for ED-based smoking cessation pathways

To translate research evidence into feasible clinical practice, EDs need post-discharge strategies that sustain motivation and continuity of care without overburdening healthcare staff. Post-discharge strategies such as automated quitline scheduling or community peer-support networks can extend the impact of brief interventions initiated in the ED, creating a structured yet low-intensity pathway toward long-term abstinence (Fig. 1).

### Automated quitline scheduling

The integration of a simple digital referral system within routine ED workflows allows clinicians to connect motivated smokers with national or regional cessation services in real time. When a patient expresses willingness to quit, the clinician completes a short electronic form, automatically populated from triage data that triggers a scheduled callback from a quitline within 48–72 h. Patients receive an immediate confirmation message with their appointment details and an option to modify or reschedule. During the appointment, cessation specialists deliver tailored counseling and recommend NRT or other quit aids (e.g., e-cigarettes or pharmacotherapy), according to patient preferences and clinical suitability. Automated text reminders over the following weeks reinforce motivation and provide links to self-monitoring tools or digital cessation apps. This system creates a reliable and scalable follow-up mechanism, ensuring that patients receive timely, individualized professional support after the ED encounter while preserving the operational flow of emergency care.

### Community “Buddy” networks

A complementary community-based model can further enhance engagement and adherence after discharge. Peer-support networks composed of ex-smokers, trained peer supporters, and community professionals such as pharmacists may offer individualized guidance on NRT, e-cigarettes, and behavioral strategies for quitting [49, 55, 61]. It should be noted that the involvement of commercial vaping retailers in clinical care pathways raises regulatory and conflict-of-interest considerations that any future implementation research must address explicitly. After brief standardized training under the supervision of cessation specialists, these peers provide evidence-based advice and ongoing encouragement through messaging platforms, phone calls, or small local meetings. Patients are typically matched to a peer within a few days of discharge, based on proximity, language, or preferences. Community partners such as pharmacies and primary care centers can serve as accessible hubs for practical assistance, product refills, and motivational support. This low-threshold, peer-led approach promotes self-efficacy, helps prevent relapse during the high-risk first month, and is particularly valuable for individuals with limited access to traditional healthcare services.

It should be noted that the evidence base for peer-supported cessation networks in ED settings remains preliminary. While conceptually promising, these models have not yet been evaluated in pragmatic trials within emergency care contexts, and should therefore be regarded as hypotheses requiring rigorous prospective evaluation before implementation at scale.

## Conclusions

Current evidence suggests that ED-initiated cessation can be effective [19, 62]: the acute illness visit creates a teachable moment, and brief interventions delivered by trained clinicians appear to increase engagement, although effect sizes remain modest and context-dependent. Pairing cognitive-behavioral support with immediate access to nicotine alternatives (NRT or an e-cigarette starter kit) appears to outperform information-only approaches at up to 12 months, though long-term effectiveness remains uncertain.

Impact, however, varies with health-system capacity and local resources. Programs depend on structured follow-up pathways to maintain quit rates and reduce relapse, a point echoed by WHO guidance [63]. This is particularly relevant where the ED is the primary or only point of care for many lower-income patients. Success hinges on embedding point-of-care treatment, reliable hand-offs to community services, and low-burden remote support.
